# Book Review

**Published:** 1901-09

**Authors:** 


**What to Do in Cases of Poisoning.** By William Murrell, M.D. Ninth Edition. Pp. 290. London: H. K. Lewis. 1900.—The eighth edition of this popular handbook was exhausted in a little over two years, and it has once more been corrected and revised. The charm of the writer's style has, no doubt, much to do with its popularity, and it must be confessed that he compresses within his tiny pages much information which can be sought for in vain from the larger text-books. In the light of recent denials of the toxic character of many salts of copper, it is interesting to find, after a reference to chronic poisoning from preserved peas, that the author holds it doubtful whether repeated small doses of copper do any harm. In support of this view he mentions his practice of giving the sulphate in nerve diseases for weeks together without any ill effects. It is probable, indeed, that many cases of supposed copper-poisoning were really due to ptomaines. The pharmacopœial substitute for chlorodyne is not correctly described in this book. It now contains $\frac{1}{11}$ grain of morphine hydrochlorate in 10 minims, and not $\frac{1}{48}$ as given on p. 111, being four times the strength of the old preparation. Many readers will think the doses of sulphate of atropine advised for hypodermic use as an antidote, such as $\frac{1}{50}$–$\frac{1}{20}$ grain, are dangerously high, in view of the many accidents recorded, and of its doubtful value in opium poisoning. Indeed, the same may be said of the half-grain injections of morphine and of pilocarpine. No reference to the use of strong alcohol in carbolic acid poisoning, and as a wash for burns from carbolic, is given, though very successful results from washing out the stomach with strong spirit have been recorded of late. Artificial respiration, too, should be included in the therapeutic measures in these cases.

**Medical Electricity.** By H. Lewis Jones, M.D., being the Third Edition of *Medical Electricity*, by W. E. Steavenson, M.D., and H. Lewis Jones, M.D. Pp. xv., 532. London: H. K. Lewis. 1900.—In this edition the subject of the utilisation of the current from the mains for medical and surgical purposes is given a separate chapter, and in an appendix there is a list of towns having a public electric-light supply, with some details of the character of the current supplied. The chapter on statical electricity has been re-written, the uses of the electric bath is treated more fully than before, and a new chapter on X-ray work has been added. This latter is one of the best summaries of the subject that we have come across, and will be of more use to the operator than many of the larger monographs that have been published, both on account of the clear explanation it gives of the phenomena observed, and also on account of the many practical hints it contains. The fact of this work having reached a third edition points to its being much read by medical electricians, and the addition of this chapter on the Röntgen rays cannot but materially enhance its value.

**"First Aid" to the Injured and Sick.** By F. J. Warwick, M.B., and A. C. Tunstall, M.D. Pp. xiii., 228. Bristol: John Wright and Co. 1901.—This will be found a useful book both by teachers and students of ambulance work. The subject is treated more fully than in the hand-books generally used, and the illustrations are numerous and well executed. The chapters devoted to elementary anatomy and physiology, poisons and their immediate treatment, and the transport of sick and wounded, are excellent. We should, however, recommend that the paragraphs relating to the treatment of electric shock be revised, in order to effect greater security to the rescuer and a better chance for the sufferer.

**Selected Researches in Pathology.** By ALEX. GUNN AULD, M.D. Pp. viii., 153. London: J. & A. Churchill. 1901. This record of further investigations by a working pathologist cannot fail to be of interest. It is a series of collected papers, previously published in various transactions, and revised for this volume. The investigations were mostly carried on in the laboratories of the Royal Colleges of Physicians and Surgeons. The most recent work is that on Pneumonia, which has but recently been finished: it relates entirely to the pneumococcus, or diplococcus lanceolatus of Fraenkel, its chemical pathology, and the action of its toxines in rabbits. Its importance is shown by the statement that "the more the organism is studied, the stronger becomes the ground for its adoption as the cause of croupous pneumonia." It is disappointing that the serum treatment of pneumonia in man does not meet with more success.

**A Text-book of Pathology.** By ALFRED STENGEL, M.D. Third Edition. Pp. 873. London and Philadelphia: W. B. Saunders & Company. 1900.—The third edition of this excellent text-book is slightly enlarged. It contains twenty-three more pages than the first edition. Two illustrations in the section upon diseases of the thyroid gland are also added. The additional matter chiefly occurs under the heading of pathologic physiology. The sections under this heading must be of considerable value to the medical student. They serve to connect the *post-mortem* room with the bedside. The book, although reaching to over 800 pages, does not seem to us to contain any information unnecessary to the student; in fact, when one feels tempted occasionally to criticise, it is not on the ground of too great fulness of statement of facts, but rather from the feeling that one would like, here and there, more detail, or some reservation placed upon a view expressed. Any such feeling, however, only shows how great is the amount of knowledge now to be mastered by the student of medicine, and the difficulty a writer who sets himself the task of preparing a text-book must find in presenting all necessary information in a concise form. It seems to us that for lucid and terse, yet comprehensive, treatment of a wide subject this book would be difficult to improve upon.

**Archives of the Roentgen Ray.** Vol. V., No. 3. March, 1901. London: Rebman Limited.—This number of the *Archives*

is specially noticeable, not only because it contains a most interesting paper by Lieut. F. Bruce on "Experiences of X-ray Work during the Siege of Ladysmith," but also because of a short account of the treatment of rodent ulcer with X-rays by Dr. Sequeira. It is to be hoped that the cures of this most obstinate disease which Dr. Sequeira seems to have obtained by the use of the X-rays will be permanent, and that other workers will meet with equal success in its employment. The reproductions of skiagrams at the end of the number are of the usual excellence we are accustomed to in this periodical.

**Annales de l'Institut de Pathologie et de Bactériologie de Bucharest.** Vol. VI. Bucharest: Imprimerie de l'État. 1898. —Victor Babes, the well-known professor of medicine at Bucharest, gives us herein an excellent instalment of very good work in many important directions. One of the most noteworthy is a contribution to the study of the etiology and pathological anatomy of pulmonary gangrene. Of great interest is the series of articles dealing with the bacteriology of hemorrhagic septicæmia, gangrene, and hepatic suppuration, conditions which according to the author may be excited by virulent cocci, pseudo-diphtheria bacilli, and saprophytic organisms introduced to the system through tuberculous lesions, or through the tonsils. These same lesions may, it is stated, also be the point of entry for the typhoid bacillus. The attempt to make some classification of the streptococci, whilst not altogether satisfactory, is a valuable addition to our means of distinguishing certain varieties of this ubiquitous organism.

**Electricity in Gynæcology.** By Richard J. Cowen. Pp. 132. London: Baillière, Tindall and Cox. 1900.—While by no means endorsing the author's encomiums on the use of electricity, a method of treatment which we have found to be disappointing in its results, we can commend this little work to those who wish to study the methods of application of the remedy in gynæcological cases. We would like to utter one word of warning to the reader: in comparing the use of caustics, the application of electricity and curettage, the author speaks of the application of an electrode to the uterine cavity as being practically safe if certain simple precautions are observed; we must plead guilty to entertaining an opinion exactly opposed to this. We presume the simple precautions— and no others are either necessary or useful beyond the careful regulation of the current—consist in asepsis. This is undoubtedly the truth, if we read the author correctly; but in our opinion, the great objection to the electric treatment is the difficulty of ensuring adequate asepsis in any one application. In curettage, on the other hand, strict asepsis will remove all the risks, and the operation is completed at one sitting. If however, the operator is not experienced in the technique of minor aseptic gynæcology the danger in either case is a very real one.

**Gynæcological Operations.** By Skene Keith, M.B. Pp. x., 118. Edinburgh: Young J. Pentland. 1900.—We can recommend this work as a lucid and reliable guide to those gynæcological operations which do not necessitate the opening of the peritoneal cavity. We notice that although the author remarks on what he considers the abuse of antiseptic methods, that his own are not by any means wanting in thoroughness. The practice which he mentions of passing graduated bougies for purposes of dilatation without anæsthesia is perhaps on the whole better avoided; it is difficult in most patients to render the vagina aseptic on account of the disagreeable character to the patient of the necessary manipulations. At the same time, everyone will agree that an unnecessary number of assistants and nurses should be avoided, in order to obviate, as much as possible, the idea of impressing on the patient that a severe operation is about to be performed. As regards the use of tents, we notice that the author does not mention the method of rendering them aseptic by soaking in absolute alcohol; this, in the case of laminaria tents, is a most useful method. We quite endorse his experience of Dudley's operation, of which he gives a most concise account. He might with advantage have described Tait's operation for perineorraphy, and Schroeder's trachelorraphy.

**The Transactions of the Edinburgh Obstetrical Society.** Vol. XXV. Edinburgh: Oliver and Boyd. 1900.—We expect good and original work from the Edinburgh Obstetrical Society, and the high standard of work we are accustomed to is fully maintained in this present volume of the *Transactions* of the Society. We would especially draw attention to two papers, by Drs. Jardine and Ballantyne, bearing further testimony to the value of saline infusions in the treatment of puerperal eclampsia, and also to Dr. Berry Hart's paper "On the Cause of the Differentiation of Connective Tissue in the Human Fœtus." But the whole volume is well worth careful reading.

**Difficult Labour.** New and Revised [Third] Edition. By G. Ernest Herman, M.B. Pp. xx., 449. London: Cassell and Company, Limited. 1901.—This book is so well known and has attained such wide appreciation that it is scarcely necessary to say more than that the author has been careful to keep all its original features. The book has been revised throughout for this edition, the most important alterations being those met with in chapters xxviii. and xxix., which are devoted to Cæsarean section and symphysiotomy. In the former chapter Dr. Herman says that when delivery is hindered by the presence of a fibroid, Cæsarean section followed by removal of the uterus with the fibroid is preferable to removal of the ovaries. As regards the production of sterility after Cæsarean section, he recommends removal of the body of the uterus, leaving the cervix, in preference to excising a piece of the tubes. Porro's operation is, he thinks, one that ought seldom to be performed, and that will soon become obsolete. Symphysiotomy, as described in this edition, is a very simple operation, the only instrument necessary being a sharp-pointed tenotomy knife; with this the symphysis is divided subcutaneously. After delivery with forceps, the two pubic bones are pressed together and kept in apposition by means of strapping and a tight binder. We note that where it is necessary to dilate the cervix Champetier de Ribes's bag is recommended in preference to the well-known Barnes's bags. The clear style and arrangement of the matter throughout the book is everything that could be desired, while the excellent diagrams will be of great assistance to the student.

**Studies from the Department of Pathology of the College of Physicians and Surgeons, Columbia University, N. Y.** Vol. VI., for the Collegiate year 1898-1899.—This volume consists of reprints of papers which have already been published in the current medical journals during 1898-1899 by workers in the above department. The more important studies issued from the department are brought together in this volume for convenience of reference. This seems a very good plan, as whilst all the papers written by the workers in the laboratory are published, only those which seem of especial importance are reprinted in this fashion. The most important article is one by Dr. James Ewing, entitled "Studies in Ganglion Cells," and occupies more than half the volume; it should be read by all who are interested in the pathology of the nervous system, as it both contains much original work and summarises the results of the most recent researches.

**Arzneiverordnungen in der Kinderpraxis.** Von Dr. H. Guttmann. Dritte Auflage. Pp. 110. Berlin: S. Karger. 1901.—This is a little book, interleaved with writing paper for notes, containing an account of those medicines which are used in the treatment of disease in children. The name of each drug is followed by a short description of its chemical and physical properties, the dose, a list of those diseases in which it may be given, and a few selected prescriptions. There is nothing quite answering to this in English, but it may be doubted whether the volume—exceedingly useful in Germany—will be of service to practitioners in England, owing to the difference between the German and English pharmacopœias.

**The Syphilis of Children in Every-day Practice.** By George Carpenter, M.D. Pp. 112. London: Baillière, Tindall and Cox. 1901.—This little book summarises the results of observations of many hundred cases of syphilis in children in a clear and interesting way. There is ample evidence in the volume that the descriptions have been written, not from general impressions derived from seeing the cases, but from careful note-taking of each individual case, and subsequent collation of the results. This is particularly evident in the sections dealing with the questions of enlargement of the spleen and liver, cranio-tabes, and Parrot's nodes. One might perhaps have wished for more pictures; but, apart from this, the book is the best account in English of infantile syphilis.

**The Physical Examination and Development of Public School Boys.** By Cecil Hawkins, M.A. Pp. 32. London: J. & A. Churchill. 1900.—This paper, which was read before the Medical Officers of Schools Association, containing an analysis of statistics based upon records of over 40,000 observations on the height, weight, rate of growth, chest girth, etc., at different ages, represents an enormous amount of labour, and the author has produced statistics and conclusions which will be of value for reference and for comparison with other existing data on such questions, which are not over-plentiful and not seldom unreliable.

**The Diseases of Children.** By Henry Ashby, M.D., and G. A. Wright, M.B. Fourth Edition. Pp. xxvi., 872. London: Longmans, Green & Co. 1899.—Dr. Ashby and Mr. Wright's book is, we believe, the best book on the diseases of children written in English, and the fourth edition has grown in matter and bulk to a rather alarming extent. New diagrams, &c., have been added, all the letterpress has been carefully revised, and, without going too much into detail, the work is as complete as it is possible to make it. No book has been so deservedly popular, and, if possible, the new edition will be more in demand than the former ones.

**Transactions of the American Pediatric Society.** Vol. X. [New York]: Reprinted from *The Archives of Pediatrics.* 1898. —Though every communication in this volume is of interest, there are two of special value; viz., the report on infantile scurvy and that on the scope and limitations of hospitals for infants, by Dr. Holt. The former of these is a carefully-compiled account of 379 cases of that rare disease that have been observed in the North continent, and contains much valuable information. We are strongly in accord with the remarks of the author of the latter.

**Diseases of the Eye.** By Henry R. Swanzy, M.B. Seventh Edition. Pp. xvii., 644. London: H. K. Lewis. 1900.—Once more we have had the pleasure of reading a fresh edition of "Swanzy's Handbook of Diseases of the Eye," and it really was pleasure. This is the seventh edition, and it shews how well the work is kept up to date that the sale of the book is as brisk as when the work first came out. As a text book for students it has no equal, and not only no equal, but, as the Americans would say, "no competitor in the same street." There is, as we said, much new matter: the chapter on X-rays, in reference to the localisation of fragments of metal in the eye, is very good, but, like all descriptions of this method, a little complicated, and hard, at first, to quite understand. We always think that one practical demonstration on the subject is much better remembered and understood than many chapters of written description. The article (for we do not like to call it merely a chapter) on "Ocular Diseases and Symptoms present in Lesions of the Brain and Spinal Cord" is the finest extant. It is divided into five parts, which shows how thoroughly the subject has been worked up. Altogether the work is a marvel, and we trust the gifted author may long be spared the energy to write many more editions. Included in the work are three tables prepared by Dr. Louis Werner for this book, which give the actions and relative values of the various mydriatics, myotics, and local anæsthetics used in ophthalmic practice.

**Transactions of the American Ophthalmological Society.** Vol. VIII., Part 3. Hartford: Published by the Society. 1899.—We have the transactions of the 35th meeting of this society before us, and find it a most instructive book and one that any society might feel proud of. One great improvement we notice in these American ophthalmological transactions is the omission of the pages and pages of wearisome accounts of vision, with correction by cylinders of ·25 dioptre. One other alteration in the way of improvement we would advise; viz., a similar omission of the question and answer style of discussion which takes place after the reading of the various papers. This may be very instructive in its way, and doubtless when listened to is interesting; but it looks rather foolish in print, and is difficult to wade through, taking up a lot of space.

The illustrations, though few this time, are very good, especially the reproductions from photomicrographs at the beginning. Among the more interesting cases we noticed was one of anterior polar cataract, illustrating a new theory of the formation of this opacity. The history of the spontaneous rupture of an eyeball, due to a sudden intraocular hemorrhage in an old glaucomatous eye, is, it seems, a unique one. There is also an article on "Uniform Test for Vision" that is worthy of perusal; but the picture of a modified perimeter is so complicated that it reminded us of nothing less than a locomotive that had burst its boiler, such as may be seen from time to time in our illustrated papers. We have had some experience of these complicated perimeters, and never knew one keep in order longer than a month—at any rate when used in hospital work. Still, this is a small matter, and does not interfere with the book being a very good one and well worth reading.

**Manual of Diseases of the Ear.** By Thomas Barr, M.D. Third Edition. Pp. xxiii., 429. Glasgow: James Maclehose and Sons. 1901. We have on a former occasion had opportunity of expressing appreciation of this excellent manual, and we now welcome the new edition, which is revised throughout and is in some parts re-written.

The most important changes will be found in the chapters dealing with the consequences of purulent diseases of the middle ear, and their operative treatment. The grafting operation recently introduced by Mr. C. A. Ballance is well described and illustrated, and the various operations of Schwartze, Stacke, Küster, and others are amply dealt with. We recommend the work as one of the best text-books on diseases of the ear.

**Diseases of the Nose and Throat.** By F. de Havilland Hall, M.D., and Herbert Tilley, M.D. Second Edition. Pp. xiii., 605. London: H. K. Lewis. 1901. Though larger than the first edition by about fifty pages, the present edition maintains the general scope and arrangement of the earlier issue. The work has been thoroughly revised and brought up to date, thus necessitating the re-writing of the sections dealing with nasal accessory sinus disease, in which progress has been so rapid and fruitful of late years.

The descriptions of the various diseases is concise and clear, and the *technique* of surgical treatment is obviously the outcome of sound and extensive practical experience.

Some of the half-tone blocks are so blurred in printing as to afford little information to beginners, and in a future edition should be printed on plate paper; but most of the newly-introduced sketches by the surgical author will convey what they are intended to illustrate more clearly than highly-finished drawings. In a practical manual it is not always possible to give the credit due to original investigators, and for the most part such references are omitted; but in one instance the authors have gone astray, for the observation on rarefying osteitis and formation of bone fragments by osteoclasts associated with nasal polypi was first described by Zuckerkandl, and has been figured for years in at least one English text-book on diseases of the nose—yet no mention of this is made, the authors attributing this pathological observation entirely to Lack's recent study on the pathology of nasal polypi, etc.

But, apart from these slight criticisms, we have pleasure in warmly commending this very excellent manual, which cannot fail to prove most acceptable to any practitioner or senior student in want of a thoroughly practical guide to diseases of the upper respiratory tract, and we congratulate both authors on the completeness and accuracy which characterises their work.

**Lectures on Nasal Obstruction.** By A. Marmaduke Sheild, M.B. Pp. x., 104. London: Rebman, Limited. 1900.—This little work contains the substance of three clinical lectures delivered at St. George's Hospital, and is illustrated by one excellent coloured plate and 27 illustrations in the text. The first lecture is on the causes and diagnosis of nasal obstruction, and the author's remarks are so sensible and so marked by evidences of experience and judicious observation that we can recommend them as well worthy of close study. The second lecture is on the treatment of nasal obstruction. There is room for much difference of opinion here; but although we by no means agree with all the recommendations of the author, yet in the main his methods are worthy of being followed. He speaks favourably of Asch's operation on deviated septum, which he has frequently performed and here briefly describes. He suggests that the celluloid splint to be afterwards worn should not be removed for fourteen days after the operation; this strikes us as an improvement on the method of changing it daily. For the treatment of hypertrophic rhinitis he prefers the galvano-cautery to the application of chromic acid as a subsequent application to small œdematous patches. There is nothing new said on the subject of adenoids, but some useful warnings are given on some possible dangers and complications of the operation for their removal, and the best way of avoiding them. The third lecture is on the treatment of nasal polypi, and this is perhaps the most important part of the work. The author believes in necrosis of the ethmoid bone as the usual cause of polypi, the proof of which he considers to lie in the feeling with the probe of bare bone far back towards the body of the sphenoid, and in the naked-eye appearance of pieces of thin paper-like bone, which after removal appear white and bare. He recommends the removal of the whole of the inferior and the anterior portion of the middle turbinate body in all severe cases of polypi, in order to allow of a thorough exploration of the nasal cavity, and in this respect he agrees with most modern operators, who now realise how difficult a matter it is to completely eradicate a chain of mucous polypi from the nose. While we do not altogether agree with the author in his pathology, yet we consider his treatment so thorough and, at the same time, so conservative that it may be well recommended and safely followed.

**Laryngeal Phthisis; or, Consumption of the Throat.** By RICHARD LAKE. Pp. 94. London: Rebman, Limited. 1901.—This small monograph is based on the observation and treatment of over 300 cases, and is mainly devoted to the description of different methods of treatment adopted by the author. A few preliminary remarks on predisposing causes are followed by a brief statement of bacteriological investigations of the nasal mucosa by Dr. J. J. S. Lucas, which tend to show that the noses of tubercular patients swarm with micro-organisms of various kinds, mainly staphylococci, streptococci, and diplococci, and only rarely reveal the presence of tubercle bacilli. In discussing the semeiology, the author lays stress on a point that is too often ignored; viz., that "the want of phthisical signs in deeply-seated disease of the lungs, and thus the erroneous impression obtained of the state of the chest in phthisical patients, with obstructive disease of the larynx, often complicates the diagnosis," and "it takes a wide experience in the examination of the chest to estimate correctly the preliminary condition in the class of case under consideration."

A series of several cases treated by the author are interesting, many showing successful results following local treatment. Six coloured plates, containing twenty-one illustrations of tubercular larynges, give a fair idea of the laryngoscopic aspects of the disease in question, and concludes a very useful addition to the practice of laryngology.

**The Practical Guide to the Public Health Acts.** By Thos. Whiteside Hime, M.D. London: Baillière, Tindall and Cox. 1901. The first edition of this book consisted of 207 pages, the new much enlarged edition numbers over 1000 pages; the printing is in good type on thin paper, and the binding is well done. We think it would have been better had the author arranged the work in two volumes, devoting one to the Public Health Acts, including the excellent epitome of legal cases which he has brought together in the appendix, while in the second volume might be given the author's views on disinfection and his numerous hints on various subjects. In reviewing the two parts we should strongly recommend as a useful book of reference the volume devoted to law, while we might criticise somewhat severely that devoted to general science. Many of the "Hints" are eminently practical, others are utopian in character, while some are evidently not based upon actual observation or practice. In the first category we should place the useful advice to young officials as to the relative value of approbation from the Central and from the Local Authority, and the "Hints on Statistics." Under "Hints as to Hospitals for Infectious Diseases," from 3,000 to 4,000 cubic feet air space is recommended for each patient; this is utopian, as also are some of the authors' recommendations for the prevention of tuberculosis, the exclusion of consumptives from certain appointments. "Hints on Cowsheds" would appear not to be based upon experience, as a width of stall of 5 feet 3 inches permits the cow to stand or lie in any direction, and so to cover herself with filth; no mention is made of the size and position of the gutter.

Under the heading "Disinfection," on page 103 of the appendix, the author makes a somewhat personal attack upon an eminent London professor, and he refers to his own report on scarlet fever and the milk supply in terms which might lead one to suppose it was advantageous to obtain milk from infected dairies; but he is very thorough in his method of dealing with infected rooms, although we do not understand how he obtains ten per cent. solutions of carbolic acid or of permanganate of potash, neither of these substances being soluble in much less than sixteen parts of water in their purest forms, while commercial carbolic acid requires considerably more.

Dr. Hime has succeeded in compiling a vast amount of information and has put it in an entertaining form. We cordially recommend this book to medical officers of health and to candidates for the diploma in public health.

**Reports and Papers of the American Public Health Association.** Vol. XXVI. Columbus: The Berlin Printing Company. 1901. —A series of papers read at the 28th meeting of the American Public Health Association, at Indianopolis, in October, 1900, constitutes the major portion of the volume. It is only necessary to turn over the pages to ascertain what are the most burning questions in sanitation, and to get valuable information on the etiology, prevention, and abolition of infections. Contagious diseases in general, the constitution and by-laws of the association, are doubtless of perennial interest to the members.

**Transactions of the American Dermatological Association.** Chicago: P. F. Pettibone and Co. 1901.—These transactions are amongst the most interesting dermatological works we are called upon to review. They are carefully and well printed, are well indexed, and, allowance made for certain peculiarities of spelling and wording, there is really nothing to find fault with.

There is much recent research brought out in the papers and subjects discussed, and we always feel we have, at least, got some new ideas.

The presidental address was given by Dr. Henry W. Stelwagon, of Philadelphia, who makes some excellent remarks about the duty and responsibility of the general physician to his cutaneous patients. The physician must acquire the special knowledge required, or, at least, put his patient in the way where it may be obtained.

The subjects discussed are many. "Loss of Hair," 300 cases, by Dr. Geo. J. Judson, of New York, although it contains little that is new regarding treatment, accentuates the views that seborrhœa of the scalp and heredity are contributing causes. Massage of the scalp, *i.e.*, judicious pinching of the scalp between the finger-ends, is recommended, of which process we can give some confirmation.

"The Prevalence of Parasitic Diseases of the Skin, and measures necessary to limit the spread" introduced by Dr. William Thomas Corlett, of Cleveland, is well worth perusal, and contains some excellent advice which the general practitioner may wisely lay to heart.

Malignant Diseases of the Skin: their nature, etiology and pathology, and treatment, by Drs. Howson, Hartzell, and Shepherd respectively, are dealt with very fully and comprehensively.

But, perhaps, the most interesting subject of all is "Blastomycosis," or "Blasto-mycetic Dermatitis." There are several excellent papers and discussions on the subject. We have long been convinced that many so-called cases of verruca necrogenica do not correspond with the usual descriptions of this disease. They rest on a sounder scientific basis when budding germs, or blastomyces, are concerned in their spread and development.

The "Report of the Committee on Statistics" is very interesting and well worth studying.

**Golden Rules of Skin Practice.** By DAVID WALSH, M.D. Pp. 102. Bristol: John Wright & Co. [1900.]—This condensed epitome contains the concentrated extract of the larger text-books. No exception can be taken to the statements made: they are well chosen and reliable: every page contains useful memoranda of things to do and things to avoid. It is difficult to learn from such a book as this, as few people can assimilate knowledge when it has been boiled down to the utmost degree of concentration.

**Chloroform.** By EDWARD LAWRIE, M.B. Pp. 120. London: J. & A. Churchill. 1901.—The physiology of this book, to use the author's own words, is based upon the assumption of the correctness of the Hyderabad experiments of 1892, and contains nothing new. In fact the book, as a whole, is not an exposition of any new theory, but merely a re-statement of an old position in the light of later observations and additional experience (p. 14).

Dr. Lawrie is an enthusiast on the subject of chloroform administration. Almost all operating surgeons in England and America have long ago convinced themselves of two facts: first, that ether is a safer anæsthetic than chloroform; and, second, that chloroform when given in an excessive dose is liable to cause sudden death by its action on the heart.

Dr. Lawrie will have none of these. He claims that Syme never had a death under chloroform, and that he (Dr. Lawrie) during a period of 8¾ years, with 17,300 administrations, has only had one death. He considers that where more frequent accidents occur the fault is in the method of administration, and the principal object of this book is to point out to students and practitioners how these accidents are to be avoided.

Whatever we may think of Dr. Lawrie's theories, we have nothing but approval for his practice. If chloroform is to be given at all, we cannot do better than follow the directions of Syme, as are here well reiterated by Dr. Lawrie. Anyone wishing to become a good chloroformist should carefully peruse the directions here given, and if he will then (contrary to Dr. Lawrie's advice) still continue to look upon chloroform as a dangerous anæsthetic, and only administer it when ether is for some reason contra-indicated, he will, in our opinion, be doing the best he can for his patients. The book is a veritable *edition de luxe*. The type is large, with ample margins, the illustrations are excellent, and the language is good, simple Saxon which can be readily understood.

**British Medical Association. Report of the Anæsthetics' Committee (appointed 1891).** Pp. 134. July, 1900.—At the Bournemouth meeting of the British Medical Association in 1891 a committee was appointed "To investigate the clinical evidence with regard to the effects of anæsthetics upon the human subject; and especially the relative safety of the various anæsthetics, the best methods of administering them, and the best methods of restoring a patient in case of threatened death." The work was left in the hands of a sub-committee, consisting of Mr. Hutchinson, Dr. Childs, Dr. Dudley Buxton, Mr. G. Eastes, Dr. Hewitt, and Mr. Rowell; and we are now presented with their report. They have come to the conclusion that chloroform is a much more dangerous anæsthetic than ether; and, contrary to the opinions of Dr. Lawrie and the Hyderabad Commission, they have found that when danger occurs under chloroform, whatever its exact nature may be, there is abundant evidence that in a large proportion of cases the symptoms that are observed are those of primary circulatory failure, and that the tendency for circulatory complications to appear increases directly with the relative amount of chloroform in the anæsthetic employed. This is quite in accordance with the observations of most administrators of anæsthetics, and no amount of experiments upon dogs either in India or elsewhere will outweigh this important clinical fact. We are sorry that the committee were unable to come to any conclusion as to the best method of restoration in case of threatened death. This is a matter that well needs further investigation. While it cannot be said that this report throws any new light upon the various anæsthetic problems, yet it gives evidence of so much pains-taking care and is so temperately worded that it will form a valuable addition to the copious literature on the subject.

**The Asphyxial Factor in Anæsthesia.** By H. BELLAMY GARDNER. Pp. 63. London: Baillière, Tindall and Cox. 1901.—We have not formed a favourable impression of this book. Though intended for the occasional administrator of an anæsthetic, it presupposes a considerable acquaintance with the art, and the facts are not given in an impressive or definite way. More than half the book is taken up by an inadequate description of the administration of anæsthetics. It would appear that the author intended to enlarge upon the fact that "a condition in which oxygen is not gaining access to the system in adequate amount is fraught with very real danger to life."

**The Journal of Hygiene.** Vol. I., No. 1. January, 1901. Cambridge: At the University Press.—We are pleased to welcome this new Exchange, published quarterly, edited by Dr. George H. F. Nuttall, in conjunction with Dr. John S. Haldane and Dr. A. Newsholme, and a lengthy list of collaborators, of world-wide distribution and reputation. The veteran leader in the science of hygiene, Sir John Simon, congratulates the editors on their work, and we may well endorse his words that "the time seems now to have come when a new journal edited by representative men of Oxford, Cambridge, and London may surely expect to escape pitfalls, and to represent in the main what shall prove to be advances in true knowledge." The first paper on the geographical distribution of anopheles in relation to the former distribution of ague in England, and one by Dr. Klein on the pathogenic microbes in milk, are of especial interest at the present moment.

**Transactions of the Antiseptic Club.** Reported by Albert Abrams. Pp. 206. New York: E. B. Treat & Company. 1900. —This book, which purports to be the record of an eccentric medical society, may be briefly described as a mixture of American humour of a third-rate order and sarcastic remarks on the foibles and ethics of medical men.

It is prefaced by a quotation from Robert Louis Stephenson, which is the best part of the book, but has little or nothing to do with what follows. There are the usual jokes about purgatives, intestines, tapeworms, etc.; but the wit is too feeble and rare to atone for the vulgarity.

There is, however, one passage which is so applicable to certain orators whom we often hear that it is worth quoting: "He can talk more and say less than anyone I ever knew. His complaint is altogether too common in medical societies. It is characterised as a diarrhœa of words and a constipation of ideas." Some of the short, introductory paragraphs to the various chapters are sensible and well put together, and the illustrations are good enough for the text.

**Burdett's Hospitals and Charities.** London: The Scientific Press (Limited). 1901.—We have once more the pleasure of welcoming the new volume of this excellent repertory. It is needless to add one word to the favourable comments in previous issues. This is not that the volume is less worthy of praise, but because it is always uniform and complete in its own field of usefulness.

**Saint Bartholomew's Hospital Reports.** Vol. XXXVI. London: Smith, Elder, & Co. 1901.—This volume contains an interesting account of the life of Sir James Paget, and a much shorter outline of the career of Sir R. Thorne Thorne. The article refers to the former as follows: "We all owe to Sir James Paget a deep and lasting debt of gratitude for the part he took in representing the profession in the eyes of the public. On every occasion and in every way he brought us great honour. For many years, wherever scientific culture, good taste, and unsullied integrity existed, he was regarded as the chief exponent of them all." Of the papers of medical interest in this volume there are many, but reference will be made only to one. Dr. C. E. Hedges writes on the ætiology of primary pleurisy. Examination was made of records of 130 cases of pleurisy with effusion, and these cases were traced after leaving the hospital in order to see what had become of them. It was found that 40 per cent. developed phthisis or some tubercular lesion within six years, and the average duration of life of those who developed phthisis was three years from the time of onset of the pleurisy. Dr. Hedges thus brings strong evidence in support of a fact noticed by others, that simple pleurisy is almost invariably tubercular. We may remark that our experience in *post-mortem* work fully bears this out. When general adhesions are found on one side of the chest, in nine cases out of ten there is scarring at the apex of the lung, or a small calcareous tuberculous focus elsewhere in the pleura. Of more importance, however, than the establishing of the tubercular origin of pleurisy, is the question of prognosis. This has been carefully elucidated by Dr. Hedges, and one of his important conclusions has already been referred to.

**Guy's Hospital Reports.** Vol. LIV. London: J. & A. Churchill. 1900.—The articles in the present volume are not of striking interest or importance. Medical literature is so superabundant at the present day that hospital reports are not of the same value which they possessed in the days of Bright and Addison. Papers on "Diseases of the Pancreas," by Dr. W. Hale White; on "Suppurative Pylephlebitis," by Dr. J. H. Bryant; and on "The Production of Gas-containing Cavities in the Internal Organs of the Body," by J. H. Bryant and W. C. C. Pakes, are among the best of the present series.

**Saint Thomas's Hospital Reports.** New Series. Vol. XXVII. London: J. & A. Churchill. 1899.—These reports give a full account of the large amount of work done in the various departments of the hospital during the year. A systematic account of the surgical technique in use at the hospital during 1899 will be of interest to surgeons. There are ten other papers of much interest, including one by Dr. W. S. Colman on "Æsculapius and his Sanctuary," and there is the usual list of old students, with their addresses, at the end of the volume.

**Glasgow Hospital Reports.** Vol. II. Glasgow: James Maclehose and Sons. 1900.—This second volume of these reports well maintains the high character of the first. It is to be hoped that they will obtain a large circle of readers, as they well deserve to do, on account of the great value to which the papers in them attain as contributions to clinical medicine and surgery. It is impossible to mention in the space at our disposal all the papers, and we must be content with calling attention to the merits of the book as a whole.

**Some Things of General Interest in the Bristol Medical Library.** By L. M. Griffiths, M.R.C.S., Honorary Librarian.—This reprint from the *Library Association Record*, June and July, 1901, for private distribution, is a paper read at the Annual Meeting of the Library Association, Bristol, September 26th, 1900. As it is of much interest to us locally, we hope to give it a more lengthened notice on another occasion.

